# A Predictive *In Vitro* Model of the Impact of Drugs with Anticholinergic Properties on Human Neuronal and Astrocytic Systems

**DOI:** 10.1371/journal.pone.0118786

**Published:** 2015-03-04

**Authors:** Elizabeth K. Woehrling, H. Rheinallt Parri, Erin H. Y. Tse, Eric J. Hill, Ian D. Maidment, G. Christopher Fox, Michael D. Coleman

**Affiliations:** 1 School of Life and Health Sciences, Aston University, Birmingham, B4 7ET, United Kingdom; 2 Aston Research Centre into Healthy Ageing (ARCHA), Aston University, Birmingham, B4 7ET, United Kingdom; 3 Norwich Medical School, Faculty of Medicine and Health Sciences, University of East Anglia, Norwich Research Park, Norwich, NR4 7TJ, United Kingdom; University of São Paulo, BRAZIL

## Abstract

The link between off-target anticholinergic effects of medications and acute cognitive impairment in older adults requires urgent investigation. We aimed to determine whether a relevant *in vitro* model may aid the identification of anticholinergic responses to drugs and the prediction of anticholinergic risk during polypharmacy. In this preliminary study we employed a co-culture of human-derived neurons and astrocytes (NT2.N/A) derived from the NT2 cell line. NT2.N/A cells possess much of the functionality of mature neurons and astrocytes, key cholinergic phenotypic markers and muscarinic acetylcholine receptors (mAChRs). The cholinergic response of NT2 astrocytes to the mAChR agonist oxotremorine was examined using the fluorescent dye fluo-4 to quantitate increases in intracellular calcium [Ca^2+^]i. Inhibition of this response by drugs classified as severe (dicycloverine, amitriptyline), moderate (cyclobenzaprine) and possible (cimetidine) on the Anticholinergic Cognitive Burden (ACB) scale, was examined after exposure to individual and pairs of compounds. Individually, dicycloverine had the most significant effect regarding inhibition of the astrocytic cholinergic response to oxotremorine, followed by amitriptyline then cyclobenzaprine and cimetidine, in agreement with the ACB scale. In combination, dicycloverine with cyclobenzaprine had the most significant effect, followed by dicycloverine with amitriptyline. The order of potency of the drugs in combination frequently disagreed with predicted ACB scores derived from summation of the individual drug scores, suggesting current scales may underestimate the effect of polypharmacy. Overall, this NT2.N/A model may be appropriate for further investigation of adverse anticholinergic effects of multiple medications, in order to inform clinical choices of suitable drug use in the elderly.

## Introduction

Use of multiple medications is common in the elderly [1,2] and polypharmacy is linked with significant reduction in mental and physical health [3,4]. Specifically, it has been estimated that as many as 70% of the frail elderly take drugs with off-target anticholinergic functionality [5]. Historically, clinical focus on anticholinergic effects has been on peripheral autonomic unwanted events, such has dizziness and dry mouth. However, cholinergic transmission at central muscarinic acetylcholine receptors (mAChR) has long been implicated in higher brain functions such as learning and memory [6]. Unsurprisingly, experimental and clinical studies have revealed a strong link between the anticholinergic properties of medications and acute cognitive impairment in older adults [1,7,8]. Latterly, these agents have also been indicated to accelerate the onset of dementia and shortening of patient lifespan, particularly in those with delirium [5,9,10].

Several approaches have been published that attempt to assess drug-induced anticholinergic burden, broadly rank anticholinergic medications and assist in the prediction of anticholinergic adverse effects. The serum anticholinergic activity (SAA) assay uses an *in vitro* radioreceptor assay to quantitate the total anticholinergic drug burden in serum via competitive inhibition by anticholinergic compounds of the binding of the potent muscarinic antagonist tritiated quinuclidinyl benzilate to solubilised mAChRs derived from rat brain [11,12]. The results are presented as picomoles of atropine equivalents per milliliter (pmol/mL), based on displacement of the benzilate compared with an atropine standard curve; values exceeding 15 pM/mL are considered high (+++), those between 5–15pM/mL medium (++) and values ranging from 0.5–5 pM/mL are viewed as low (+) [12]. In clinical practice, several expert-based models which aim to score the anticholinergic drug burden of an individual have also been developed. Of these, the Anticholinergic Cognitive Burden (ACB) scale was developed with reference to frequently prescribed medications, clinical experiences (medical literature related to anticholinergic adverse effects and determine rates of anticholinergic adverse effects compared with placebo), expert opinion and evidence of *in vitro* affinity for muscarinic receptors. The ACB scale then ranked medications on a 4-point scale (0—no anticholinergic evidence; 1—drugs with SAA but no known clinically relevant anticholinergic effects; 2/3—drugs with established clinically relevant cognitive anticholinergic effects of increasing degree) [13].

Whilst peripheral SAA has been used to detect overall muscarinic anticholinergic properties of various medications, as well as in the prediction and management of patient risk, the association with central and cognitive cholinergic function remains unclear. Indeed, the association of anticholinergic burden assessments and electroencephalogram (EEG) parameters in a patient group with high delirium risk was investigated and it was concluded that SAA levels did not reflect cerebral cholinergic function [14]. As all the expert-based score models are partly based upon the *in vitro* detection of SAA, the limitations of SAA and the drug scales overlap. In addition, the choice of methods used to measure changes in anticholinergic burden and cognitive decline has recently been found to have a significant effect on the results of causal association studies [15]. In all cases the overall score for an individual patient is considered to be the sum of points of their medications [7,16].

The current indirect *in vitro* and clinical methods, though complex and expensive, may not be sufficiently informative to predict anticholinergic risk, especially from combinations of medications. Therefore, we considered the reliable identification of anticholinergic impact to require urgent investigation. It has previously been suggested that the study of anticholinergic pharmacological mechanisms using a biological model would inform further predictive studies [17]. Clearly the ideal *in vitro* biological assay would be an advanced form of human neuronal tissue, with a functional neuronal and astrocytic network, which was capable of cholinergic transmission. As such, we employed the NTERA-2 (NT2) cell line, established from a human male germ cell carcinoma. These cells are capable of differentiating into post-mitotic neurons and astrocytes, designated NT2.N [18] and NT2.A [19] cells respectively. NT2 neurons exhibit virtually all the functional competencies of central nervous system (CNS) neurons and they have cholinergic, GABAergic and glutamate receptor and neurotransmitter systems [20]. These cells generate action potentials, as well as sustaining neurotransmitter release and response [21,22], as well as expressing functional synapses [23]. They also express the high voltage activated L, N, P/Q and R calcium channel currents [24] and calcium activated BK channels which are involved in neuronal hyperpolarisation following action potential firing [25]. Our group has extensive experience of these cells, demonstrating that NT2 neuronal networks signal to NT2 astrocytes in co-culture (NT2.N/A), and that astrocytic networks communicate via gap junction-mediated and gliotransmitter signalling [22].

In order for an *in vitro* model to be applicable for the pharmacological study of anticholinergic effects, it must express functional muscarinic acetylcholine receptors (mAChR). This family of five G Protein-coupled receptor subtypes (m1–5) are widely distributed on multiple organs and tissues and are critical to the maintenance of central and peripheral cholinergic neurotransmission [26]. All five mAChR subtypes have been identified in the brain and their regional expression varies between species [27]. Regarding the intrinsic suitability of the NT2.N/A co-culture model, functional mAChRs have previously been identified in NT2.N cells [28]. Additionally, cultured astrocytes exhibit a wide variety of functional neurotransmitter receptors, including all five mAChRs [29].

Astrocytes are now known to respond to signals from cholinergic neurons, with the activation of cellular mAChRs being coupled to an increase in astrocytic intracellular calcium [Ca^2+^]_i_ and with their blockage having the opposite effect [28]. Information processing and brain storage was classically thought to rely solely on neurons. However, it has been demonstrated that astrocytic [Ca^2+^]_i_ increase as a result of cholinergic transmission is crucial to the mechanisms of learning and memory [30]. Thus it was decided to focus on the mAChR- induced calcium responses of NT2 astrocytes in order to develop an *in vitro* predictive model of the anticholinergic impact of polypharmacy on the CNS. In this study, the confirmation of cholinergic activity in NT2.N/A cultures was achieved via calcium imaging by exposing them to oxotremorine (a nonselective muscarinic acetylcholine receptor agonist) and using the fluorescent calcium dye fluo-4 to detect Ca^2+^ mediated network responses in terms of changes to astrocytic [Ca^2+^]_i_. Blockage of these responses by increasing concentrations of clinically relevant drugs with anticholinergic functionality (Table 1) was examined and the resulting dose-response curves used to rank these drugs and combinations thereof for anticholinergic potential, in comparison with the existing ACB scale as well as with some published SAA values for amitriptyline and dicycloverine.

**Table 1:** The drugs employed in the present investigation with their respective Anticholinergic Cognitive Burden (ACB) scores and Serum Anticholinergic Activity (SAA) scores.

Drug	Use	ACB score	SAA Score (pM/mL)
Amitriptyline Hydrochloride	Antidepressant Pain relief	3 (severe)	>15 (High)
Dicycloverine Hydrochloride	Anticholinergic antispasmodic	3 (severe)	>15 (High)
Cyclobenzaprine Hydrochloride	Antispasmodic	2 (moderate)	
Cimetidine Hydrochloride	H_2_ antagonist	1 (possible)	

## Methods

### 1. Materials

The NTERA-2 clone D1 (NT2.D1; Human Caucasian pluripotent embryonal carcinoma) cells (European Collection of Cell Cultures Catalogue Number 01071221) were kindly donated by Professor P. W. Andrews (University of Sheffield, UK). Cell culture products were from Invitrogen (Paisley, UK) and all other reagents and chemicals were from Sigma (Poole, UK), unless otherwise stated.

### 2. Generation of NT2.N/A co-cultures

Production of the NT2.N/A co-cultures was based on previous plating methods ([19, 31] with modifications as previously described by ourselves [32,33]. Briefly, NT2.D1 cells (2 × 10^6^ cells/75 cm^2^ flask) were treated (unpassaged, to allow a build-up of layers of cells) for 4 weeks using DMEM high glucose medium (DMEM-HG), changed twice weekly and supplemented with 10% (v/v) foetal bovine serum (FBS), 100 units/ml penicillin, 100 μg/ml streptomycin and 1 × 10^-5^ M all-trans retinoic acid (RA). Differentiated cells were replated (sub-cultured 1:3; replate #1) and incubated for two days, then dislodged and replated onto CellBIND (Corning, Netherlands) 6-well plates (2.5 × 10^6^ cells/well, replate #2) and treated with mitotic inhibitors for 28 days; 0.1 μM cytosine arabinoside (for the first 7 days only), 3 μM fluorodeoxyuridine and 5 μM uridine. No further passaging took place as cells had reached a post-mitotic stage. For some experiments, 12 mm poly-D-lysine (PDL)/Laminin Corning Biocoat round coverslips (VWR International, Lutterworth, UK) were placed in the wells prior to replate #2.

### 3. Characterisation of the NT2.N/A cells

**3.1 Immunocytochemistry and fluorescent image acquisition**. NT2.N/A cells cultured on coverslips were fixed using 4% (v/v) paraformaldehyde at 4°C for 1 h and then blocked with 0.2% Triton X-100 in PBS and 5% (w/v) bovine serum albumin (BSA) for 2 h at room temperature. Coverslips were incubated with mouse anti-neuronal class β-tubulin-III (Clone TUJ1, Covance, UK, 1:500), Mouse anti-Glial Fibrillary acidic protein (Clone GA5, Millipore 1:500) or mouse anti- anti-choline acetyltransferase (ChAT: clone 1.B3.9B3, Millipore, 1:400). Coverslips were washed and incubated with donkey anti-mouse rhodamine (Jackson ImmunoResearch, Europe, 1:200) or rabbit anti-Mouse FITC (Jackson ImmunoResearch, 1:200) for 1 h at room temperature. After washing the coverslips were mounted on slides using Prolong Gold Antifade Mountant (Life Technologies, UK) before analysis using the Leica SP2 fluorescent microscope with the x10 dry/oil objective and the green filter (495nm) and red filter (546nm) cubes for FITC and Rhodamine visualisation respectively. Images were analysed using the LAS AD Lite software (Leica Microsystems, Milton Keynes, UK) or a Zeiss LSM 510 META confocal laser-scanning microscope. Cells were imaged at optimal intervals throughout the entire depth of the section and acquired as a Z-stack.

**3.2 Western blotting**. The protein extraction and western blotting procedure was carried out as previously described by us [34], using 10 μg protein/well, in duplicate. For immunoblotting, after blocking the blots were incubated with primary antibodies overnight at 4°C; mouse anti-acetylcholinesterase (AChE, clone AE-1, Millipore, Watford, UK, 1:500), mouse (ChAT, clone 334008, RnDSystems, Abingdon, UK, 1:500). After washing the blots were incubated for 1 hr at RT with horse anti-mouse IgG-horseradish peroxidase (HRP) (7076, Cell signalling, MA, USA, 1:500) secondary antibody. After washing, protein-antibody binding was detected on film (Hyperfilm ECL; Amersham) using enhanced chemiluminescence (ECL Western blotting analysis system; Amersham Pharmacia).

**3.3 Muscarinic acetylcholine receptor (mAChR) sandwich ELISAs**. NT2.N/A cells were frozen at-20°C overnight, followed by three freeze/thaw cycles, where each time the cells were thawed to room temperature then frozen at-20°C. (~40 mins per cycle). During each cycle, the cells were examined under the microscope to ensure full freeze/defrost had occurred in order to achieve full lysis. Following centrifugation (5000 g, 4°C, 10 min), protein in the supernatant was quantitated using the Pierce BCA protein assay kit (Fisher Scientific, Loughborough, UK). Quantitative analysis of all 5 muscarinic acetylcholine receptors (mAChRs) in the cell supernatant was performed using human mAChR Sandwich ELISA systems exactly as the manufacturer’s protocol (2bscientific, Upper Heyford, UK). A standard curve (2000–31.25 pg ml^-1^ purified mAChR with sample diluent only acting as a negative control), was run alongside and used to calculate the mean concentration of muscarinic receptors in the samples (pg ml^-1^) and the protein concentration of the lysate used to determine the concentration in fmol mg^-1^ protein. Each ELISA was performed on 3 independent occasions and data are presented as the mean ± standard error of the mean (SEM).

### 4. Calcium Imaging

**4.1 Solutions and chemicals**. Binding of agonist drugs to cholinergic muscarinic receptors (mAChR) stimulates release of Ca^2+^ from membrane bound stores, causing a rise in intracellular free calcium [Ca^2+^]_i_ which is measurable using the fluorescent calcium dye, fluo-4 (Molecular Probes, Eugene, OR). Initially the mAChR functionality of the NT2 astrocytic cells was established by exposure of the cells to increasing concentrations of the non-specific mAChR agonist oxotremorine. Secondly, inhibition of the astrocytic response to oxotremorine was examined. Four drugs on the ACB ranking scale were chosen [7] and examined singly (Table 1) and in six combinations of two (amitriptyline with dicycloverine, amitriptyline with cyclobenzaprine and so on).

**Fig 1:**
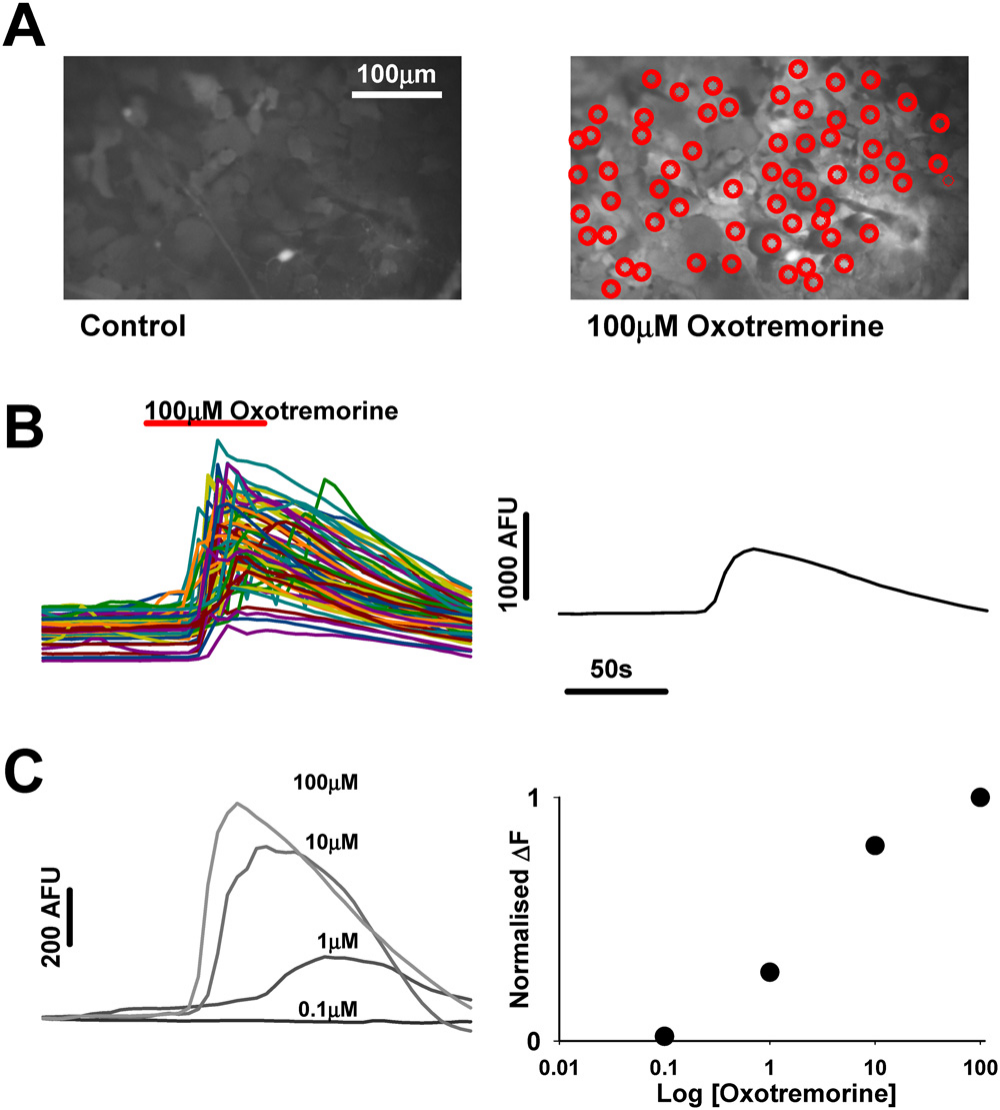
Determination of agonist effect on NT2A cultures. A. Images of field of astrocytes before (left) and during (right) 100μM Oxotremorine application. Red circles indicate regions of interest (ROI’s) from which fluorescence (AFU-arbitrary fluorescence units) over time are sampled. B. Plot of fluorescence over time showing the effect of oxotremorine on the circled astrocytes in A. Plot on the right displays the average of the astrocyte fluorescence over time. C. Average of fluorescence traces from the same ROI’s to 0.1,1,10 and 100μM oxotremorine. Plot on the right displays the normalised change in fluorescence from the traces on the right to give the dose response relationship.

**4.2 Cell perfusion: mAChR agonist—oxotremorine**. Oxotremorine in base medium was applied to the coverslip via a perfusion pump for 1 min at a rate of 1.5 ml min^-1^ in the following concentrations (μM) 0.1, 1, 10 and 100, with 4 min perfusion with base medium only between each drug concentration. The concentration of oxotremorine required to elicit the half maximal effect (EC_50_) regarding increase in astrocytic intracellular calcium was then determined as outlined in Fig. 1 (oxotremorine EC_50_ = 2.5 μM, see results section 3.2).

**4.3 cell perfusion: mAChR antagonists—amitriptyline, cyclobenzaprine, cimetidine, dicycloverine**. The cells were first perfused with the established EC_50_ of oxotremorine (2.5 μM) in base medium for 1 min followed by 4 min perfusion with base medium only, in order to establish the maximal agonist effect at this concentration. Next, anticholinergic drugs together with oxotremorine at 2.5 μM were applied via a perfusion pump for 1 min at a rate of 1.5 ml min^-1^ in the following concentrations (μM): 0.005, 0.05, 0.5, 5, 50 followed by 4 min perfusion with base medium only. However, prior to the challenge with oxotremorine (together with each anticholinergic drug concentration), the cells were perfused for 5 min with the relevant antagonist concentration only (during which time image recording did not take place). The concentration of each antagonist required to inhibit the response of the cells to the EC_50_ concentration of oxotremorine to 50% regarding the increase in astrocytic intracellular calcium (IC_50_), was then determined.

**4.4 Measurement of evoked astrocytic [Ca** ^**2+**^] **increases**. NT2.N/A cells on coverslips were loaded with 5 μM fluo-4 and 0.01% pluronic acid in DMEM-HG without phenol red, for 60 min at 37°C. Following washing the coverslip was placed in the calcium imaging recording chamber (Luigs and Neumann, Germany) fitted to a Nikon FN1 upright microscope, with filter cubes obtained from Chroma (Chroma VT, USA). The cells were perfused as described in section 4.2 and the fluo-4 was excited using LEDs at excitation and emission wavelengths of 488nm and 516 nm respectively. Images of areas of 444 μm×341μm were routinely acquired every 5 s for 5 min with a ×20 objective lens (NA = 0.8) using an ORCA ER CCD camera (Hamamatsu) and analysed using Simple PCI software (Compix Hamamatsu, Digital Pixel, UK). For each coverslip at least 80 astrocytes in each image were designated as a region of interest (ROI) for analysis (Fig. 1). The data produced were transferred to a spreadsheet and the average fluorescence intensity of the ROIs at each time-point calculated.

**4.5 Determining the EC**_**50**_ **for oxotremorine**. The highest mean fluorescence (F_max_) of the ROI (Fig. 1) was designated the maximal response to the oxotremorine agonist and from this the lowest mean baseline fluorescence (F_0_) was subtracted (F_max_-F_0_ = ΔF). The results were expressed as ΔF for each oxotremorine concentration as a percentage of ΔF in the presence of the highest concentration of oxotremorine used, which was considered to elicit the maximal response (100μM; set at 100%). From this a concentration-response curve was plotted and the EC_50_ for oxotremorine in the NT2 astrocytes determined using Graphpad Prism software.

**4.6 Determining the IC**_**50**_ **s for the mAChR antagonists**. Again, the highest mean fluorescence (F_max_) was designated the maximal response to the oxotremorine agonist and from this the lowest mean baseline fluorescence (F_0_) was subtracted (F_max_-F_0_ = ΔF). This was done for each antagonist concentration and the results were expressed as ΔF for 2.5 μM oxotremorine in the presence of each antagonist concentration as a percentage of ΔF in the presence of 2.5 μM oxotremorine only (set at 100%). From this concentration-response curves were plotted and the IC_50_ for each antagonist or combination determined using Graphpad Prism software.

### 5. Statistical analysis

Each perfusion experiment was performed using 3 individual coverslips of cells and data are presented as the mean ± standard error of the mean (SEM). The mean EC_50_ ± SEM (oxotremorine) or IC_50_ ± SEM (antagonist drugs) of the three independent experiments were determined following non-linear regression curve fit analysis using Graphpad Prism. One-way ANOVA followed by the Tukey multiple comparisons post-test was used to determine significant differences between the IC_50_ values.

## Results

### 1. Characterisation of the NT2.N/A cell cultures

**Fig 2:**
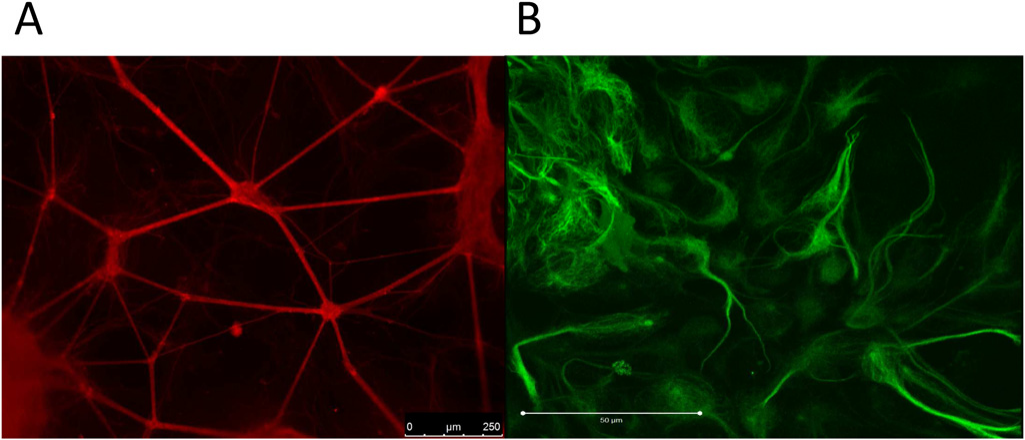
Immunofluorescence images of NT2 neurons and astrocytes. A) Bundles of β-tubulin III positive NT2 neurons interconnected with neurites (stained red) and B) GFAP positive NT2 astrocytes (stained green).

**1.1 Immunocytochemistry—β-tubulin III and GFAP**. Specific immunostaining for the cell-type specific intracellular markers β-tubulin III and GFAP distinguished with secondary antibodies conjugated to different fluorochromes (β-tubulin positive neurons stained red, GFAP positive astrocytes stained green) was used to produce images of the NT2 neuronal network (Fig. 2A) and associated NT2 astrocytes (Fig. 2B). The neuronal cell bodies formed small, evenly dispersed aggregations (50–100 μm) with linking bundles of neurites, which covered the entire surface of the coverslip. The network appears supported by a monolayer of astrocytic cells, also covering the whole of the coverslip and clustered around the aggregations of neuronal cell bodies, both beneath and between neurite outgrowths. Many of the GFAP positive cells demonstrate the stellate morphology with short processes expected of human astrocytes.

**Fig 3:**
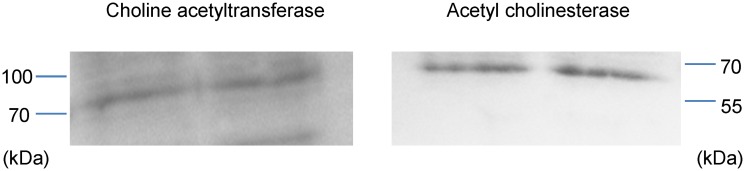
Representative western blot analysis of NT2.N/A cell lysate proteins. The cholinergic markers choline acetyltransferase (ChAT) and acetylcholinesterase (AChE) were detected in NT2.N/A cell lysates (10 μg/well).

**1.2 Immunoblotting—ChAT and AChE**. Immunoblotting was carried out using the anti-ChAT clone 334008 and mouse anti-AChE clone AE-1 antibodies to examine for the presence of the phenotypic cholinergic markers ChAT and AChE in the NT2.N/A cell lysate proteins. The anti-ChAT antibody cl.334008 recognises both the ChAT isoform 1 (ChAT-69; cytoplasmic) and isoform 2 (ChAT-82; nuclear), however only one band was observed in our experiments (Fig. 3). Comparison with molecular markers run alongside the samples revealed this protein’s molecular weight to be consistent with isoform 1 (69 kDa, RnDSystems, UK). Such soluble forms are responsible for 80–90% of the enzyme’s activity. A single band was also observed using the AChE antibody (Fig. 3) and comparison with the molecular markers confirmed the molecular weight of the protein to be approximately 68 kDa, which is consistent with the manufacturer’s predicted band size (Millipore, UK).

**Fig 4:**
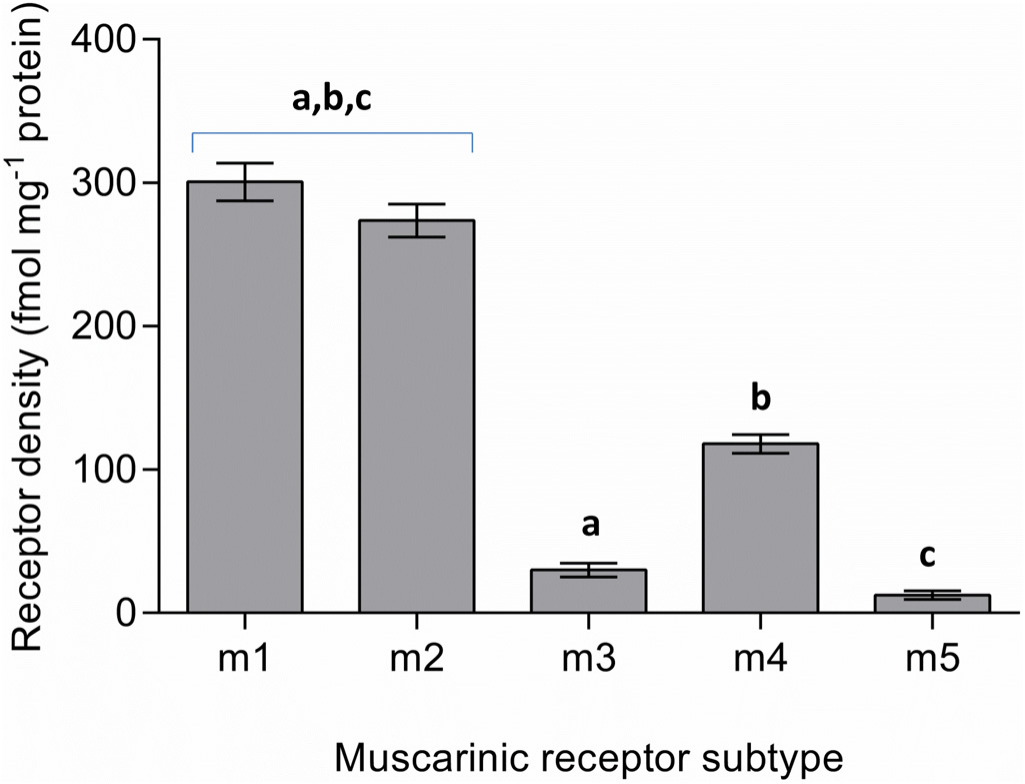
Quantitative analysis of muscarinic acetylcholine receptors (mAChR): receptor concentration (fmol mg-1 protein) values were determined using sandwich ELISAs for each subtype. Bars represent the mean ± SEM of the means from three separate experiments: Values marked ‘a’, ‘b’ or ‘c’ are significantly different from each other (P<0.001). Additionally, values for m3 and m5 are significantly different from those of m4 (P<0.001), whilst values for m3 and m5 are also significantly different from each other (P<0.05)

**1.3 Muscarinic acetylcholine receptor (mAChR) quantitation by ELISA**. Five muscarinic receptor subtypes have been identified in the brain and quantitative analysis of all mAChRs (m1–5) in the NT2.N/A lysed cell supernatant was performed via specific human sandwich enzyme immunoassay. From the respective calibration curves run alongside the samples and their protein content, the concentration of muscarinic receptors in the NT2.N/A supernatant was calculated (fmol mg^-1^ protein) and determined to be 300.8 ± 313.1 (m1), 273.8 ± 11.5 (m2), 30 ± 4.8 (m3), 118 ± 6.6 (m4) and 12.5 ± 3 (m5; Fig. 4). This translates to approximate relative percentage (%) values of mAChRs in NT2.N/A cells of 41 (m1), 37 (m2), 4 (m3), 16 (m4) and 2 (m5). Thus, the predominant receptor subtypes in our neuronal-astrocytic co-culture seemed to be the ACh m1 and m2 receptors, whose levels were not considered significantly different, followed by the m4 receptor (P < 0.001), with a significantly lower level of expression of both the m3 and m5 receptors (P < 0.001).

### 2. Response to cholinergic and anticholinergic drugs

**Fig 5:**
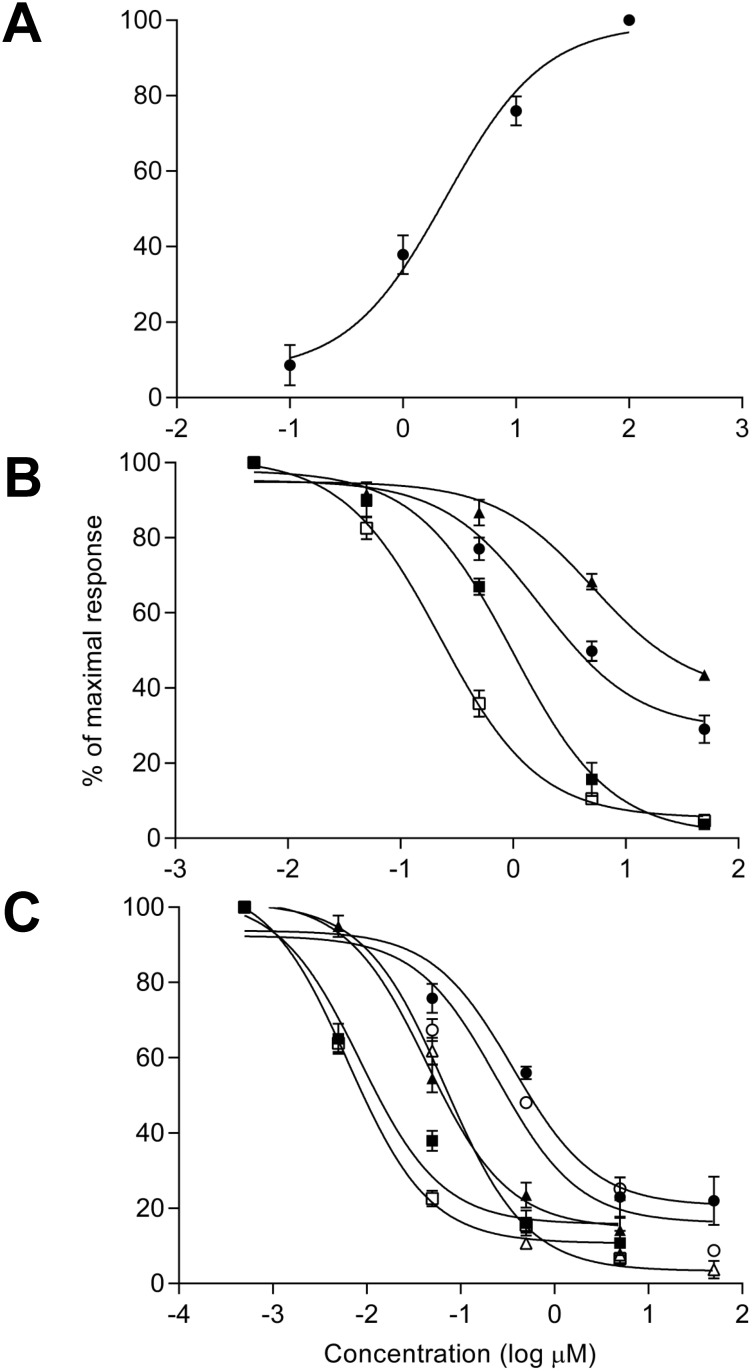
Effects of anticholinergic agents on NT2.N/A culture responses to the cholinergic agonist oxotremorine. A) Effect of perfusion of NT2.N/A cultures with oxotremorine for 1 min on release of Ca^2+^ from astrocytic membrane bound stores; showing a sigmoidal log concentration-response curve for [Ca^2+^]_i_ increase in the NT2 astrocytes. B) Inhibition of the astrocytic response to the EC_50_ concentration of oxotremorine by single anticholinergic drugs, Sigmoidal log concentration—response curves for inhibition of the [Ca^2+^]_i_ increase in the NT2 astrocytes in response to increasing concentrations of antagonist; dicycloverine (□ open square), amitriptyline (■ solid square), cyclobenzaprine (● solid circle) and cimetidine (▲ solid triangle). C) Comparison of the inhibition of the astrocytic response to the EC_50_ concentration of oxotremorine by anticholinergic drugs in combination. Sigmoidal log concentration—response curves for inhibition of the [Ca^2+^]_i_ increase in the NT2 astrocytes in response to increasing concentrations of antagonists in combination; amitriptyline and cimetidine (● solid circle), cyclobenzaprine and cimetidine (○ open circle), amitriptyline and cyclobenzaprine (▲ solid triangle), dicycloverine and cimetidine (Δ open triangle), dicycloverine and amitriptyline (■ solid square) and dicycloverine and cyclobenzaprine (□ open square). All values determined using the fluorescent calcium dye fluo-4. Results are expressed as a percentage of the 100 μM (A) and 2.5 μM (B & C) oxotremorine values (designated the maximal response) following background correction. Data points represent the mean ± SEM of the means from three separate experiments.

**2.1 Oxotremorine**. Release of Ca^2+^ from astrocytic membrane bound stores in response to the non-specific mAChR agonist oxotremorine was measured using the fluorescent calcium dye, fluo-4 (Fig. 1). As can be seen in Fig. 5, increasing concentrations of oxotremorine resulted in a sigmoidal-response in respect of increased Ca^2+^ release. The concentration of oxotremorine required to elicit the half maximal response (EC_50_) regarding increase in astrocytic intracellular calcium [Ca^2+^]_i_ was determined to be 2.45 ± 0.12 μM and thus a value of 2.5 μM was used for the antagonist experiments (see below).

**2.2 Inhibition of the astrocytic response to oxotremorine by anticholinergic drugs: perfusion with single compounds**. The concentration of oxotremorine required to elicit the half maximal response (EC_50_) regarding increase in astrocytic [Ca^2+^]_i_ was determined to be 2.5 μM (see section 3.2.1) Following this, the inhibition of the astrocytic calcium response to 2.5 μM oxotremorine by mAChR antagonists was examined. As can be seen in Fig. 5, increasing concentrations of all four antagonists (dicycloverine, amitriptyline, cyclobenzaprine and cimetidine) were associated with a progressive decrease in Ca^2+^ release from astrocytic membrane bound stores. This confirms that all four antagonists were capable of inhibiting the astrocytic cholinergic response to oxotremorine. IC_50_ values (Table 2) indicated that dicycloverine was significantly the most potent antagonist (0.27 ± 0.046 μM), followed by amitriptyline (1.0 ± 0.1 μM, P < 0.05), followed by cyclobenzaprine (4.7 ± 0.6 μM; P < 0.05) and with cimetidine having the least significant effect (21.7 ± 2.3 μM; P < 0.001). This order of potency of the single compounds compared favourably with their respective ACB scores (Table 2).

**Table 2:** Summary of the IC_50_ (μM) values obtained for each antagonist drug with their respective Anticholinergic Cognitive Burden (ACB) and Serum Anticholinergic Activity (SAA) scores.

Drug	IC_50_ ± SEM (μM)	ACB score	SAA score pM/mL
Dicycloverine	0.27 ± 0.046 ^a^ *	3	>15 (high)
Amitriptyline	1.0 ± 0.1 ^b^ *	3	>15 (high)
Cyclobenzaprine	4.7 ± 0.6 ^c^ ***	2	
Cimetidine	21.7 ± 2.3	1	

Values represent the mean IC_50_ values (μM) ± SEM (n = 3) for each drug.

Compared IC_50_s;

a dicycloverine vs amitriptyline,

b amitriptyline vs cyclobenzaprine and

c cyclobenzaprine vs cimetidine.

* P < 0.05,

** P < 0.01,

*** P < 0.001

**2.3 Perfusion with anticholinergic drugs in combination**. As can be seen in Fig. 5, increasing concentrations of all six antagonist combinations (see Table 3) were also associated with a progressive decrease in Ca^2+^ release from astrocytic membrane bound stores. This confirms that all six antagonist combinations were capable of inhibiting the astrocytic cholinergic response to oxotremorine. IC_50_ values (Table 3) indicating that dicycloverine with cyclobenzaprine was the most potent antagonist combination (10 ± 1.3 nM), followed by dicycloverine with amitriptyline (22 ± 2.7 nM; P < 0.05). The effects of amitriptyline with cyclobenzaprine (65 ± 9.4 nM) and dicycloverine with cimetidine (66 ± 3.4 nM) were not considered significantly different. The least potent antagonist combinations were cyclobenzaprine with cimetidine (560 ± 30 nM; P < 0.001), followed by amitriptyline with cimetidine (670 ± 45 nM; P < 0.05). In the majority of cases, the order of potency of the drugs in combination did not agree with their respective ‘predicted’ ACB scores (Table 3), which were derived from the sum of scores of the individual drugs (Table 2).

**Table 3:** Summary of the IC_50_ (nM) values obtained for the antagonist drugs in combinations as indicated, along with their ‘predicted’ ACB scores.

Drug	IC_50_ ± SEM (nM)	‘Predicted’ ACB score
Dicycloverine/Cyclobenzaprine	10 ± 1.3 ^a^ *	5
Dicycloverine/Amitriptyline	22 ± 2.7 ^b^ *	6
Amitriptyline/Cyclobenzaprine	65 ± 9.4	5
Dicycloverine/Cimetidine	66 ± 3.4 ^c^ ***	4
Cyclobenzaprine/Cimetidine	560 ± 30 ^d^ *	3
Amitriptyline/Cimetidine	670 ± 45	4

Values represent the mean IC_50_ values (n = 3), ± SEM (nM) for each combination of two drugs.

Compared IC_50_s;

a dicycloverine/cyclobenzaprine vs dicycloverine/amitriptyline,

b dicycloverine/amitriptyline vs amitriptyline/cyclobenzaprine,

c dicycloverine/cimetidine vs cyclobenzaprine/cimetidine and

d cyclobenzaprine/cimetidine vs amitriptyline/cimetidine.

* P < 0.05,

** P < 0.01,

*** P < 0.001

‘Predicted’ ACB score from linear addition of individual drug ACB scores (Table 2).

The IC_50_s obtained show the combinations of drugs to all be significantly more inhibitory (P<0.001) compared with those of their single counterparts. For example dicycloverine with amitriptyline was 12-fold and 45-fold more potent than dicycloverine or amitriptyline alone, respectively. The exception was amitriptyline alone, compared with amitriptyline with cimetidine, the IC_50_s for which were not considered to differ significantly.

## Discussion

The core objective of this study was to develop a relevant human-based and functional neuronal/astrocytic network to enable the prediction of the impact of combinations of drugs with anticholinergic effects, as channelled through mAChRs. It is essential that any model employed possesses the cholinergic phenotype. Acetycholine is crucial for cholinergic neuronal communication. ChAT catalyses the production of the acetylcholine and AChE hydrolyses it and together they serve as markers for functional cholinergic neurons [35]. The NT2.N/A model consists of a network of β-tubulin III positive neuronal cells in co-culture with GFAP positive astrocytes and we confirmed the presence of ChAT and AChE in the model via immunoblotting. It has been estimated that 1:3 differentiated NT2 neurons in pure cultures present the molecules phenotypic of cholinergic neurotransmission [36–39]. As our model encompasses both neurons and astrocytes, it would be interesting in future to examine the effects of the presence of NT2.A cells on cholinergic NT2.N numbers. Indeed, AChE and ChAT are also actively secreted by astrocytes [40,41].

The levels of all five specific mAChRs in the NT2.N/A co-culture were determined quantitatively and it was found that the predominant subtypes were m1 and m2, followed by m4 and with negligible amounts of m3 and m5. In humans, m1, m2 and m4 mAChRs are the most abundant subtypes in the forebrain. The basal forebrain is considered to be responsible for the major cholinergic output of the CNS and the basal ganglia has been implicated with voluntary movement, learning, memory and sleep. The hippocampus plays a key role in the consolidation of information from short to long term memory and spatial navigation. The m2 receptor is the dominant receptor type in the basal forebrain, with the m4 subtype being predominant in the basal ganglia. In the hippocampus, m1 receptors are thought to account for 35–60%, with m2 and m4 each accounting for 15–25% of the subtypes in this region [6]. The cerebral cortex is the outermost layered structure of neural tissue which plays a key role in memory, attention, perception, thought, language, and consciousness and contains a mixed population of m1 and m2 sites [42]. Our preliminary results indicate a similar proportion of muscarinic receptors to be expressed by NT2.N/A cells to that of these areas of the brain receiving of cholinergic input, which supports the applicability of the model. Quantitative analysis of mAChRs in pure cultures of NT2 neuronal cells has previously found the m3 subtype to be the most abundant, followed by m2 and with negligible levels of m1 and m4 [43]. The discrepancy between these findings and ours may be due to the presence of astrocytes in our NT2.N/A co-culture. Astrocytes are essential for normal neuronal function which adds to the complexity and relevance of the model and indeed, astrocytes themselves possess functional mAChRs [44].

Neuronal response to neurotransmitters following feedback via an increase in astrocytic intracellular free calcium [Ca^2+^]_i_, modulates CNS activity [45] and we have previously shown NT2 astrocytes to respond to exogenously applied neurotransmitters with calcium elevation [22]. In the current study, we determined the calcium response of NT2 astrocytes to the non-selective mAChR agonist oxotremorine and the inhibition of this [Ca^2+^]_i_ increase by a number of commonly used anticholinergic medications on the ACB scale, alone and in combination. When the four drugs were studied singly, their potency in terms of their IC_50_s corresponded with their ACB score rankings, with dicycloverine and amitriptyline showing the greatest impact and cimetidine the least (Table 2). To place our preliminary findings in the context of likely anticholinergic burden, they must be considered alongside clinical determinations of human CNS drug concentrations. For instance, the pharmacological impact of centrally active drugs such as antidepressants is directly related to their free drug concentrations. It was previously assumed that plasma protein and CNS drug binding were of similar magnitude, although it has been established that this is not the case for many centrally active drugs [46,47] and brain drug binding is considerably higher than that of plasma. With amitriptyline for example, values for plasma concentrations (360–900 nM) are in clear contrast with values predicted for that of unbound drug with the human brain (14–35 nM) after clinical doses [48]. In the present study, our values for the impact of amitriptyline alone in terms of its anticholinergic inhibitory effects (IC_50_ 1000 nM; Table 2) were considerably higher than the unbound human clinical concentrations. Likewise, plasma steady state values for dicycloverine of ~280 nM (40 mg tablet) [49] and cyclobenzaprine of ~100 nM (30 mg tablet) [50] have been measured following a single dose in healthy volunteers. It can be expected therefore, that the unbound concentrations within the brain would be considerably lower than our values for the impact of dicycloverine (270 nM) and cyclobenzaprine (4700 nM) alone in terms of their anticholinergic potential. However, it is well established that the elderly often exhibit considerably higher drug plasma and therefore potentially higher unbound brain levels, compared with younger adults. With cyclobenzaprine, plasma levels in the elderly are double those of younger subjects [51] and this is the case with many CNS-active drugs [52]. There is a lack of appropriate pharmacokinetic studies regarding dicycloverine but it is conceivable that unbound brain levels in the elderly may approach the IC_50_ of 270 nM measured for this drug in the present study, as this was the most potent of the medications examined with respect to anticholinergic effects. Additionally, our IC_50_ values for a number of the drugs in combination also approach human clinical concentrations (as described above), for example dicycloverine plus cyclobenzaprine (IC_50_ 10 nM; Table 3) or dicycloverine plus amitriptyline (22 nM). This is of great relevance as up to 40% of the elderly in care homes are being treated with anti-depressants [53] and other anticholinergic medications [54] concurrently.

A key observation of this study is how our preliminary findings contrast with the way anticholinergic drug impact is currently measured and considered clinically. As previously mentioned, expert-based anticholinergic score models for patients are calculated from the summation of each drug’s score [7,16]. However, in Table 3, the rank order of anticholinergic potential of the drugs in combination as determined from their IC_50_ values, doesn’t always correlate with the simple summation ‘predictive’ combined ACB score. For example, the combination of dicycloverine with cyclobenzaprine (IC_50_ 10 nM) which carries a ‘predictive’ ACB score of 5 was significantly more potent than the combination of dicycloverine with amitriptyline (22 nM; P < 0.01) which carries a ‘predictive’ ACB score of 6. It is likely that this discrepancy is at least partly accounted for by the synergistic effects we have shown in this report. Indeed, we have shown a combination of dicycloverine and cyclobenzaprine to be approximately 27- and 470-fold more potent respectively than either drug singly (tables 2 and 3). Additionally, the combination of amitriptyline and cyclobenzaprine was approximately 15- and 72-fold more potent respectively than either drug singly, which suggests that there may be a synergistic effect in terms of the anticholinergic impact of these drugs in combination, at concentrations which are clinically relevant. Our findings can be seen in the context of a recent report, where the idea that the anticholinergic effects following cumulative drug exposure are linearly additive, has previously been flagged as an oversimplification [55]. The aetiology of such synergy is likely to be complex and operate at several levels. These may include effects at presynaptic muscarinic receptors, such as those involved with atropine-mediated synergistic pesticide toxicity in insect systems, whose muscarinic receptors are similar to those of vertebrates [56]. Presynaptic nicotinic cholinergic receptors have also been implicated in human neuromuscular cholinergic antagonist synergy, although receptor conformational changes induced by multiple antagonist binding may be the more plausible explanation [57].

Thus, though complex and expensive, current clinical assessment of anticholinergic burden and the subsequent ranking of medications with anticholinergic functionality may not be sufficiently informative to predict anticholinergic risk from combinations of medications likely used by an elderly population, as the summative approach employed by current scales may under-estimate the effect of polypharmacy. We have shown that our human-derived NT2.N/A co-cultures possess the required cellular functionality to act as an appropriate biological model with which to investigate anticholinergic pharmacological mechanisms *in vitro*. Unlike the rodent source of muscarinic receptors employed in the SAA assay, the NT2.N/A cell are human-derived and appear to express the relevant mAChR subtypes and in the appropriate proportions. Though preliminary, the data obtained in this study suggests the NT2.N/A model may prove suitable for inclusion in a framework for the ranking of drugs alone and in combinations for anticholinergic potency, in order to inform further predictive clinical studies and assist in the prediction of anticholinergic adverse effects. The model may also be suitable for the future determination of muscarinic receptor binding affinities, which will be valuable as part of future estimations of anticholinergic impact in the human brain.

Another vital component of human research into anticholinergic impact in the future would be the *in vitro* modelling of the role of the blood brain barrier (BBB) in the systemic anticholinergic drug penetration into the CNS, as well as that of any metabolites formed by the liver or other biotransforming organs. Clinically, anticholinergic agents differ in their abilities to enter the brain and there is evidence that the brain microvessel robustness and permeability is impaired by age and infirmity [58, 59] The NT2.N/A co-cultures could be combined with advanced stem cell-derived human models [60] of the BBB in bioreactor systems to further enhance clinical predictability in terms of anticholinergic drug impact.

Future work will aim to examine a greater number of drugs on the ACB scale, alone and in combination of two or more, as this is well within the capabilities of the flexible perfusion system employed. Consultation with experts in the various fields of the primary care of the elderly, such as geriatric psychiatrists, will reveal the most relevant drug combinations worthy of study that exhibit the most anticholinergic effects at clinically relevant doses and CNS free drug concentrations, with the overall aim of establishing a reliably predictive model. Whilst there are a large potential number of possible anticholinergic drug combinations used in clinical practice to investigate, such efforts would be rewarded in terms of the discovery of which new and older drug combinations cause the most damage to the CNS cholinergic health of the elderly.
